# The Melting Diagram of Protein Solutions and Its Thermodynamic Interpretation

**DOI:** 10.3390/ijms19113571

**Published:** 2018-11-12

**Authors:** Kálmán Tompa, Mónika Bokor, Péter Tompa

**Affiliations:** 1 Institute for Solid State Physics and Optics, Wigner RCP of the HAS, Konkoly-Thege út 29-33, H-1121 Budapest, Hungary; tompa.kalman@wigner.mta.hu (K.T.); bokor.monika@wigner.mta.hu (M.B.); 2 Institute of Enzymology, Research Centre for Natural Sciences of the HAS, Magyar Tudósok körút. 27, H-1117 Budapest, Hungary; 3 VIB Center for Structural Biology, Vrije Universiteit Brussel, Building E, Pleinlaan 2, 1050 Brussel, Belgium

**Keywords:** protein, hydration, wide-line ^1^H NMR

## Abstract

Here we present a novel method for the characterization of the hydration of protein solutions based on measuring and evaluating two-component wide-line ^1^H NMR signals. We also provide a description of key elements of the procedure conceived for the thermodynamic interpretation of such results. These interdependent experimental and theoretical treatments provide direct experimental insight into the potential energy surface of proteins. The utility of our approach is demonstrated through the examples of two proteins of distinct structural classes: the globular, structured ubiquitin; and the intrinsically disordered ERD10 (early response to dehydration 10). We provide a detailed analysis and interpretation of data recorded earlier by cooling and slowly warming the protein solutions through thermal equilibrium states. We introduce and use order parameters that can be thus derived to characterize the distribution of potential energy barriers inhibiting the movement of water molecules bound to the surface of the protein. Our results enable a quantitative description of the ratio of ordered and disordered parts of proteins, and of the energy relations of protein–water bonds in aqueous solutions of the proteins.

## 1. Introduction

Wide-line ^1^H NMR is an accepted method to delineate the structures of hydrogen-containing molecules determined primarily by X-ray and, to a lesser extent, by neutron-scattering. This way, it can provide information on the location and structural environment of hydrogen atoms in proteins. It has a unique capability, on the other hand, in the direct observation of translational and rotational movements of molecules in the condensed phase.

NMR characteristics of aqueous solutions rapidly frozen and then slowly thawed through equilibrium thermal states provide direct information on the immobile and partially or fully mobile parts of the molecules. We have previously reviewed relevant features of this approach in our works “Hydrogen skeleton, mobility and protein architecture” [1] and “Studying molecular motions in solid states by NMR” [2].

Based on these studies, we state that molecular motions in the sample result in narrowing of the wide-line NMR spectrum. This phenomenon is known as motional narrowing in the literature [3]. Our goal is to advance from this observation to arrive at the thermodynamic characterization of protein systems.

**Figure 1:**
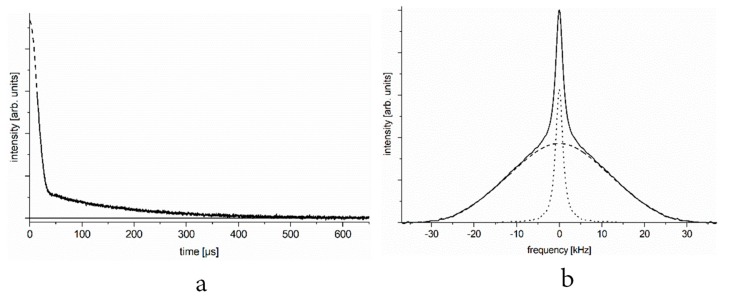
Free induction decay (FID, panel (**a**)) and spectrum (panel (**b**)) of a motionally two-state spin system. (We focus on the slow component of the FID, the initial part of which is lost in the dead time of the spectrometer, marked by dashed line, and can be disregarded).

In Figure 1a, we show the typical ^1^H NMR free-induction decay (FID) signal of a set of spins containing proton–proton pairs of different mobilities. In Figure 1b, we present the deduced NMR spectrum. Similar FID signals and NMR spectra are observed at certain temperatures when studying the aqueous solution of a protein that contains hydrogen pairs.

Time domain (Figure 1a) and frequency or energy domain (Figure 1b) representation of the spectra are linked through Fourier transformation, yet it may be useful to consider both, as they provide information on different practical utilities. The amplitude of the FID signal (even considering its slow component) extrapolated to time zero gives the number of relevant nuclei (spins) through nuclear magnetization. The amplitude of response to the 90° radiofrequency pulse is proportional to the relevant *x*-*y* component of nuclear magnetization that is further proportional to *M*_0_ ≈ (*nB*_0_)⁄*T*, in which *B*_0_ is the constant magnetic induction, *T* is the absolute temperature, and *n* is the number of resonant nuclei (in our case, it equals the number of protons in water). On the other hand, the width of the spectrum gives direct information on the motional characteristics of proton spin pairs. In a system of two components (e.g., one that contains both mobile and immobile spin-pairs), it is important to have direct information on both parameters.

It is questionable whether such a simple approach can give significant novel information on the dynamics of a complex system, such as a protein and its environment in an aqueous solution. The independent measurement over a broad temperature range of the two parameters of the slow FID component (FID amplitude extrapolated to *t* = 0 and the spectral width) is debatable.

Therefore, here we address the behavior of the slow-FID, and the narrow-spectral component. Our working hypothesis (that we already partially proved) is that the narrow spectral component comes from water molecules bound to the protein, termed bound water-molecules [4]. One may ask a range of relevant questions about their number, their strength of binding to the protein vs. the neighboring water molecules, and about their potential field following molecular changes of the protein, etc. Similar questions can also be asked for the broad-spectrum component, which we have already addressed before [1,2].

**Figure 2:**
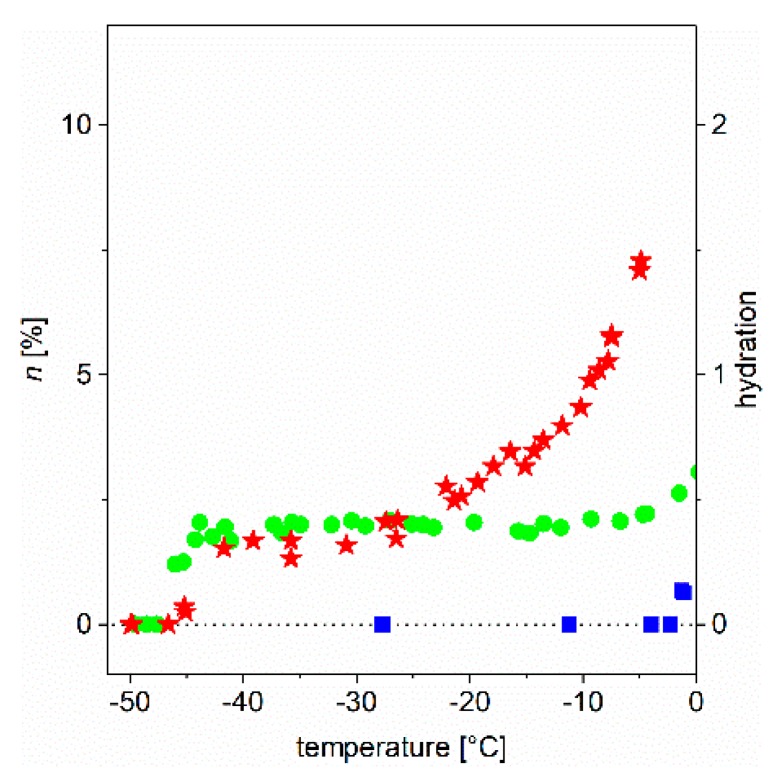
“Old fashioned” melting diagrams, i.e., the total number of mobile water molecules (through protons) normalized to the total number of water molecules, as a function of temperature (blue squares: bulk water, green circles: ubiquitin, red stars: ERD10 proteins in aqueous solutions). The data are given for 50 mg/mL protein concentration.

In earlier studies [5,6,7,8], we have addressed in detail the behavior of globular and intrinsically disordered proteins (IDPs) in aqueous solutions and provided an initial and partial interpretation of experimental observations. As relevant examples, we refer to results with proteins, such as ubiquitin, bovine serum albumin, α-synuclein (and its point mutants), calpastatin, ERD10 (early response to dehydration 10), and lysozyme. Here, we demonstrate our point by focusing on two proteins, ubiquitin (Ubq) and ERD10, as they have been thoroughly studied earlier; one (Ubq) is a globular/structured protein and the other (ERD10) is intrinsically disordered, i.e., they are representatives of these distinct structural classes. We show the temperature dependence of the slowly-decaying component of the FID extrapolated to *t* = 0 (which gives directly the ratio of relevant mobile water protons). We show the observed behavior in the form of a melting diagram (*MD*). In Figure 2, we show the *MD* of three studied systems (bulk water and the aqueous solution of two proteins, ubiquitin and ERD10) in the usual °C scale.

The melting process of inhomogeneous systems (such as the protein solutions we study), basically differs from the first-order phase transition of homogeneous, single component material, such as the melting of ice at a given transition temperature.

We consider melting as the process of the beginning of movement of a component of the mixture (such as a bound water-molecule, or a fragment of the protein of high symmetry, e.g., a methyl group or other terminal moiety), in which either translation or rotation begins. In our case, these (individual) events of initial movements show a temperature distribution characteristic of the given molecule, and the derived *MD*s link the well-defined, directly measurable NMR characteristics with atomic/molecular motions.

These characteristics can thus also give direct information on molecular interactions. The water molecules associated with the protein molecule constitute an integral part of the system. Thus, their nuclei, rather than large energy particles applied in scattering techniques (such as X-ray crystallography), monitor the potential energy surface of the protein as built-in probes. In our previous works [5,6,7,8], however, we only drew qualitative conclusions from the *MD*s.

These were as follows. In aqueous solutions, melting (that is, beginning of molecular motions) of protein-bound water molecules begins at a much lower temperature than the melting of bulk ice. Each protein has a unique *MD* (individual profile or fingerprint) that results from its individual thermodynamic characteristics. The *MD* of globular and ID proteins vastly differ. They can be characterized by temperature-independent FID amplitudes—a plateau (globular protein)—or they can lack a plateau (IDP) or can have a plateau of small temperature extension (partly IDP).

### 1.1. Energetic Interpretation of Melting Diagrams

We have made significant advances in several respects of interpreting our results [9,10] since we last addressed these questions [5,6,7,8]. Key steps are detailed in chapters 4–6 of ref [9]; here, we add a new element and summarize these steps in more detail, following the logical order of the application.

As a reminder, we are following the beginning of the movement—probably the rotation—of water molecules bound to the surface of the protein, by observing motional narrowing in wide-line ^1^H NMR spectroscopy. For the first time in the field—following the seminal work of Kittel and Kroemer [11]—we introduced the concept of fundamental temperature, *T*_f_, and also introduced here the idea suggested by Waugh and Fedin [12] for connecting the thermal excitation energy, *V*_0_, in which molecular motions begin with the temperature, *T*, as *V*_0_ = constant × *T*.

In some detail, the key steps taken are as follows.

#### 1.1.1. Fundamental Temperature

As a first step, we introduced the use of the scale of fundamental temperature, i.e., thermal excitation energy scale, *T*_f_, and its version normalized to the melting point of ice, *T*_fn_. By definition, *T*_f_ = *k*_B_*T*, in which *k*_B_ = 1.381·10^−16^ erg/K (*k*_B_ = 1.381·10^−23^ J/K) is the Boltzmann constant, and *T* is the absolute temperature in K. We can also use the equation of *T*_f_ = *RT*, in which *R* = 8.317 J/mol·K, the universal gas constant. If we need a dimensionless scale, it is expedient to use the normalized fundamental temperature scale, *T*_fn_, nor malized to the melting temperature of bulk water formally as *T*_fn_ = *k*_B_·*T*/(*k*_B_ × 273.15) = *T*/273.15. This way, it becomes possible to characterize the events of the beginning of molecular motion on an energy scale.

#### 1.1.2. Energy Scale and the Heterogeneity of the Protein Surface

As a next step, we invoked the formula of Waugh and Fedin, after the improvement of placing it on a fundamental temperature scale of the right dimension. The formula can then be used for aqueous solutions. The equation at atomic/molecular level is

*E*_0a_ [erg] = *ck*_B_*T* [erg], (1a)

or applied to molar quantities it is

*E*_0m_ [kJ/mol] = *cRT* [kJ/mol]. (1b)

In these equations, *c* is a dimensionless quantity, i.e., a number, the value of which was determined by applying Equation (1b) to the melting of bulk ice, considering the melting heat of ice (6.01 kJ/mol [13]). The fundamental temperature equivalent with 273.15 K is *T*_f_ = *RT* = 2.272 kJ/mol. In Equation (1b), the *c* proportionality constant is 2.65. When comparing Equation (1a) with the energy pertaining to one degree of freedom by the equation of equipartition (1/2 *k*_B_*T*), we may deduce the degree of freedom of a water molecule as 5.3, which seems to be in the right range for a rotating (and not translating) electric water dipole.

**Figure 3:**
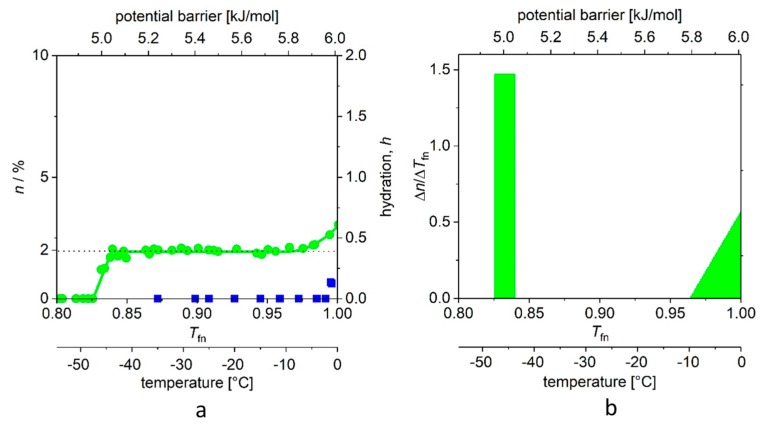
(**a**) Melting diagram (*MD*, green circles) of ubiquitin dissolved in double distilled water and that of frozen water under identical conditions (blue squares). (**b**) *DMD* curves (that is, the potential barrier distribution of protein–water bonds). There is no reliable measured data in the range −1–0 °C (0.995–1.00 *T*_fn_). The data are given for 50 mg/mL protein concentration.

**Figure 4:**
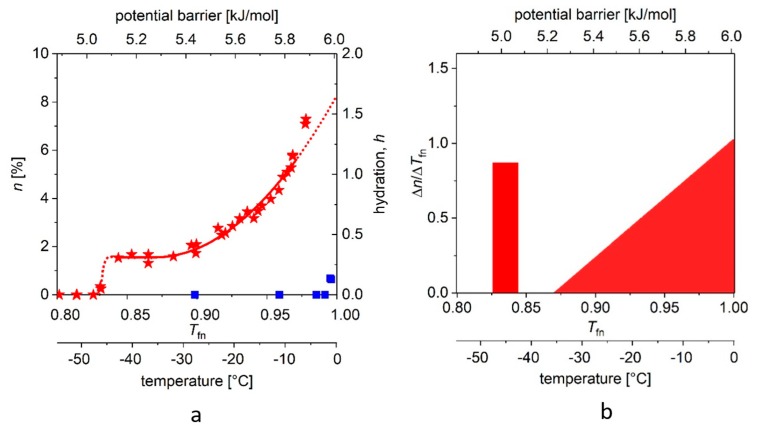
(**a**) The melting diagram (*MD*, red stars) of ERD10 dissolved in double-distilled water and the melting curve (blue squares) of the solvent (water). (**b**) *DMD* curves are shown (that is, the potential barrier distribution of protein–water bonds). There is no reliable measured data in the range −1–0 °C (0.995–1.00 *T*_fn_). The data are given for 50 mg/mL protein concentration.

In addition, we introduced dynamic parameters for the quantitative characterization of the ordered/disordered state of protein molecules, which goes beyond their static structural description. Before formalizing the definitions, let us take a look at Figure 2 (and for details, Figure 3 and Figure 4). There is a marked difference between the globular and intrinsically disordered proteins. On the melting diagram of the globular protein Ubq one can see a broad, temperature- (or excitation energy-) independent region (plateau). On the other hand, the plateau of the IDP ERD10 is significantly smaller. (A similar behavior was also seen for other proteins [5,6,7,8,9,10]). Significantly more information is provided by the initial (*T*_fno_) and the ending temperature (*T*_fne_) values of the plateau. The region between these two temperatures shows homogeneous bond (potential energy barrier) distribution, whereas the region above *T*_fne_ shows a heterogeneity in terms of protein–water-bond energy distribution. After this introduction, the following quantities can be defined.

Heterogeneity ratio, *HeR*. According to our observations [5,6,7,8,9,10] and the literature quoted therein, protein molecules can be characterized and categorized by the homogeneity/heterogeneity of the energy distribution of water binding. The basis of the classification is the measurement of the ratio, for which we suggest the relation

*HeR* = (1 − *T*_fne_)/(1 − *T*_fno_), (2)

in which (1 − *T*_fne_) and (1 − *T*_fno_) give the measured distances from the melting point of ice. These values can be easily read from the novel *MD*s. *HeR* is 1 (one) for systems showing heterogeneous water binding (lacking a plateau) and 0 (zero) for homogeneous binding systems (e.g., bulk water), and is between 0 and 1 for partially heterogeneous systems. *HeR* therefore gives the order parameter type specification for what extent of the surface of the protein molecule can be regarded as showing heterogeneous potential energy distribution (disordered) in terms of water binding. It must be emphasized that this correlation measures the heterogeneity ratio based on the comparison of the extent of the two possible regions and does not measure the number of actual protein–water bonds in them.

#### 1.1.3. An Analytical Description of *n*

The introduction of fundamental temperature or energy scale makes it possible to describe *MD* by power series in the form

*n* = *A* + *B*(*T*_fn_ − *T*_fn1_) + *C*(T_fn_ − *T*_fn2_)^2^ + … (3)

That is, we can define the total number of water molecules, *n*, that are moving at a given thermal energy (temperature), as well as the change of *MD* on a normalized fundamental energy scale, i.e., the differential form of melting diagram, *DMD*

∆*n*/∆*T*_fn_ = *B* + 2*C*(*T*_fn_ − *T*_fn2_) + …, (4)

which defines the number of water molecules that begin to move at the given excitation energy. *T*_fnx_ (with *x* = 1, 2, …, *n*) is fitting parameter in Equations (3) and (4), in which *x* is equal to the exponent in each term (in the other terms too, with *n* ≥ 3 not given here in detail). The present form of equations calls attention to the validity of any term in a given temperature range. It should be emphasized that all quantities and coefficients are dimensionless in these formulae.

Number of protein–water bonds, *HeR*_n_. We can make a statement about the homogeneity/heterogeneity of bonds (potential barriers) if we ask about the exact number of protein–water bonds in the given excitation energy range. Parameters that fit the power series provide the answer. In the simplest cases (including, in our experience, aqueous solutions with distilled water), in which there is only a wider heterogeneous range in *MD*, the number of water bonds in the heterogeneous region depends on the number of fitting members, *B*/(1 2212 *T*_fne_), and 2*C*/(1 − *T*_fne_); if both, then it depends on the sum of the two members. As simplification of the determination of the number of protein–water bonds (the degree of hydration), it can be directly read from the *DMD*s, i.e., the value or the sum of the areas colored in the figures enter (in principle, the definite integrals within the region *T*_fne_ to *T*_fn_ ≈ 1). Let *n*_ho_ be the number of water molecules in the first hydrate shell and *n*_he_ the total number of water molecules in the entire heterogeneous region. In this case, the second relation suggested for the ratio of heterogeneity is

*HeR*_n_ = *n*_he_/(*n*_he_ + *n*_ho_). (5)

The value of *n*_ho_ (approximately) is given by the area of the rectangle at the lowest excitation energy region, whereas *n*_he_ in our case is given by the areas of triangles (in general, those described by members of higher exponents; see Figure 3 and Figure 4).

The numbers in the equation can be measured directly based on *MD*. *n*_ho_ can be determined with high accuracy as the average of all *n* points measured on the plateau, and (*n*_he_ + *n*_ho_) as an approximate value by the *n* value reliably measured at a temperature close to the highest temperature, *T*_fn_ ≈ 1. The process has a self-checking potential and thus improves the reliability of the data.

The measure of heterogeneity is *HeM*. We suggested [9] to introduce this as the parameter

*HeM* = (*B* + 2*C*)/(1 − *T*_fne_). (6)

This relationship is also correct in terms of dimensions, and the *HeM* value is generally a positive number. Its value is zero for proteins of almost equipotential molecular surface, so it can be considered as a quasi-order parameter. The denominator, (1 − *T*_fne_) designates the energy range in which there are varying protein–water bonds (of heterogeneous distribution), and *B* + 2*C* (going till the second term of non-zero exponent) is the number of bonds within this range. The fraction is thus a kind of slope of the *MD* function; its values cannot be limited to the range of 0 to 1, just like for the tangent function. Non-heterogeneously binding proteins, such as globular proteins by our experience, have a *HeM* value, by definition, which applies to the region above the plateau. It is not unfounded to suggest that there is a similar dynamic difference in the hydrogen mobility (*HM* [1]) and *HeM* parameters, i.e., in the mobility of all proton–proton pairs, and in the degree of heterogeneity of protein–water bonds.

In the power series of *n* Equation (3), we only went till the first two members of non-zero exponent (which is enough to interpret the results presented in most of our examples). Heterogeneity and homogeneity can be observed in both the nature and the magnitude of the respective potentials and their distance dependence. Variants of theoretical possibilities are found in the literature [14,15,16,17].

The determination of the *MD* function and its differential form that can also be described analytically allows for the unique and individual mapping of the energy distribution of the potential barriers that inhibit the motion of water molecules bound to the protein. Using the elements required for the interpretation of measured *MD*s we have introduced, the purpose of our present work can be easily formulated. Specifically, it is a deeper, thermodynamic interpretation of our results. The examples that illustrate this statement are presented through the analysis of the *MD* of the globular standard protein ubiquitin, and the intrinsically disordered ERD10.

## 2. Results and Discussion

In Figure 3 and Figure 4, we show the *MD*s determined for the two proteins, dissolved in double-distilled water, with a panel (a) showing measurements on reference water too, and panel (b) the derived curves, *DMD*s (that is ∆*n*/∆*T*_fn_ the potential distribution of protein–water bonds). The information on the origin of the samples, the measuring equipment, and the details of the measurements is described in our above-mentioned articles and in book chapters [4,5].

Perhaps it is not unnecessary to repeat that the amplitude value of the slow component of the measured FID signal extrapolated to *t* = 0 gives directly the number *n* of resonant protons (i.e., the protein-bound water molecules), whereas the temperature dependence of *MD* gives the dependence of *n* on thermal excitation energy.

The information can be read from Figure 3a and Figure 4a as follows. Bulk water (blue squares) show the microscopic image of the ice-water phase transition. What would we expect of an absolute pure water sample of infinite size (in theory, one having a periodic boundary condition)? A single step of infinite slope at *T*_fn_ = 1.00 and *E*_a_ = 6.01 kJ/mol excitation energy (at 0.00 °C), in which all four bonds of the water molecule in the tetrahedral bond symmetry environment “melt” simultaneously. Instead moving water molecules are detected already below 0 °C. There are several reasons for this. The sample is not of infinite size, and the environment of the water molecules on the surface of the small sample is not the same as of those in the bulk environment. Secondly, the sample is not of absolute purity, so the environment of pollutions is not the same as in the clean environment. Third, the temperature of the sample in the measurement can be controlled and determined with limited accuracy only, especially at 0 °C.

In Figure 3a, the “melting point” (−46 (1) °C (for definition of error, see Table 1) of the aqueous solution of ubiquitin shows the thermal energy investment (Δ*Q*) that is required to start to move the water molecules that are bound to the protein. The steep step (with a narrow, ≈0.01 kJ/mol energy range) shows that there are water molecules in the first hydrate shell that are bound almost identically. It is a reasonable approximation to consider these energies nearly the same, and the relevant molecular surface equipotential. This potential field of nearly identical elements resembles the feature of the H-bridges [16,17,18,19] and is largely different from strongly distant dependent potentials (the variants can be find in the text-books [16,17,18,19]). The number of protein–water bonds in the actual region is given by the area of the rectangle. As a self-check, the same quantity can be more accurately determined from the average of all *n* points on the *MD* plateau.

The next wide region is the plateau. (This region begins at *T*_fno_ = 0.832 (4), in Figure 3b, in which the value of ∆*n*/∆*T*_fn_ is zero). The plateau carries very important information. No new water molecules begin to move in this excitation energy region, because there are no water molecules that are bound by corresponding energy to the protein. We can suggest that the H-bridges here, which link the bulk of the protein molecule to a globule. Thus, this can be an ancestral form of a higher order structure, which is represented not only by geometry but also by a bonding network of a certain energy. The heat invested within the plateau region does not start to move new water molecules; rather, it increases the specific heat, and the rotational speed of already rotating water molecules, as we have seen in a previous work on differential scanning calorimetry (DSC) measurements and data interpretation [7]. (Based on this interpretation, these statements can be made to be more accurate, which we intend to do in a short notice.)

*T*_fne_ is the end of the plateau, and here begins the energy region where there are binding energies close to the binding energy of water–water bonds, presumably on parts of the protein molecule that are better exposed to water. In principle, the temperature dependence of *n* can be described by the higher exponents of the power function; the quadratic member was sufficient in this case. All data are available; we summarize the values and the order parameters introduced by us in Table 1.

In Figure 4a, we show the “melting point” (approximately −42 (2) °C) for ERD10 (red stars). We also repeat the above procedure for ERD10 with different parameters. The steep step (with narrow, ≈0.01 kJ/mole energy range) shows the presence of water molecules of nearly identical binding energy in the first hydrate shell, but this region is followed by a plateau, which is significantly narrower than that observed in the case of globular proteins. We then observe a phase of continuous rise in *MD*, which can be well approximated by the quadratic (or even higher) component of the summation. A much larger part of the molecular surface is exposed to water than in the case of ubiquitin, i.e., about 69–77% of the protein molecule can be described as disordered. The range (1 − *T*_fne_) of energy barriers inhibiting water movements (which can be defined as disorder) is more than three times broader than for the selected globular protein.

Figure 3b and Figure 4b depict the changes (differential quotient) of the mobile water fractions by normalized functional temperature, i.e., they are the graphical representations of Equation (4). As outlined, the bars at low temperature (around −45 °C) correspond to the relatively high differential quotient values describing the first few data points greater than zero. The fraction of mobile hydration water increases here within a few degrees to the level of *n*(*E*_a,o_) or *A* while the first mobile hydration layer forms, which gives the high differential quotient values.

A comparison of *HeM* values (analogous with the tangent function) shows that in globular proteins the realization of the two extreme values, conditions in the first hydrate shell and water-water bonding, are very close. For ERD10, a much wider distribution of potential energy barriers is characteristic of structural disorder. The typical data are summarized in Table 1. The ordered/disordered state of the two protein molecules only approximates the ideal limiting values, *HeR* = 0 and *HeR* = 1.

The reality of the *n*_ho_ number of protein–water bonds in the homogeneous binding energy region (in other words, in the first hydrate shell) is better appreciated by reference to our knowledge of the hydration of protein-forming amino acids [18]. The sum of the numbers of the possible H-bridges of ubiquitin molecule gives 211. According to our measurements, the number of water molecules bound in the first hydration shell by similar binding energies is *n*_ho_ = 226 (3).

The summation of possible H-bridges within ERD10 yields 986. According to our measurements, the number of water molecules bound in the first hydration shell by similar binding energies is *n*_ho_ = 514 (13). The difference between measured and estimated values is unsurprising, especially in light of the good agreement found for ubiquitin. It is reasonable to ask the question whether approximately half of the H-bridges does not link with other water molecules, but realize some other type of bond.

Among the quantities given in Table 1, it is necessary to emphasize the determination of the relative number of bonds that fall into the heterogeneous region (*n*_he_/(*n*_he_ + *n*_ho_)). The result is surprising if one is thinking in terms of a globular protein molecule, because for ubiquitin, the protein is in contact with an additional *n*_he_ > 102 (33) water molecules, which is approximately 36% of all bound water-molecules. The bonds of these water molecules are dominated by water–protein bonds, which are close in energy to the of water–water bonds. In the case of ERD10, the protein surface is in contact with an additional *n*_he_ > 2200 (220) water molecules, which is approximately 73% of all bound water-molecules. In the bonds of the latter water-molecules, water–protein bonds similar to water–water bonds dominate with a substantially wider energy distribution for this initially disordered protein.

It is maybe unnecessary to emphasize that the values we suggest are derived from direct measurements, i.e., they do not rely on assuming any hypothesis or model! They allow to determine the number of first-neighbor water molecules per amino acid (*n*_ho_/amino acid), which is 226/76 ≅ 3.0 for UBQ and 514/260 ≅ 2.0 for ERD10. The round value within an error of 1% is surprising, as well as the close match of 2.0 with other values observed for other globular proteins (casein, lysozyme, and BSA, to be published). 

Therefore, the measured number of bound water-molecules for ubiquitin is 328 (30). Molecular dynamics simulation estimation from the literature [19] gives a value of 379. For ERD10, the numbers per protein molecule is 2714 (263) (measured) and 881 (estimated by molecular dynamics simulation [19]). The difference between the two proteins and the reverse ratio raise many questions about the nature of protein–water bonds that are still difficult to answer.

## 3. Materials and Experimental Methods

### 3.1. Selection of Proteins

We have selected these proteins, as we and others have collected ample evidence for their function depending on their particular structural class, which is folded and intrinsically disordered. Ubiquitin (UBQ) is a small, 76-amino acid globular protein that is found ubiquitously in the cells of all eukaryotic organisms, carrying out basic and indispensable functions in regulating protein function [20]. That is, proteins targeted for degradation are covalently modified by a mono- or poly-ubiquitin chain and are directed for degradation by the 26S proteasome, whereas other proteins labeled with a mono-ubiquitin chain enter regulatory interactions in transcription regulation, for example. 

ERD10 [21], on the other hand, is a plant dehydrin that has its cellular protection function strictly linked with structural disorder [22]. Its length is 260 amino acids; it is structurally disordered by a broad range of biophysical techniques, and it functions by protecting the structural integrity of client proteins under the conditions of dehydration and other stresses.

### 3.2. Expression and Purification of Proteins

Lyophilized ubiquitin (UBQ) was obtained from Sigma Chemical (St. Louis, MO, USA), whereas plant late embryogenesis abundant protein early response to dehydration 10 (ERD10, UniProt P42759) was produced via recombinant expression in *Escherichia coli* BL21(DE3) Star expression strain and purified as described previously [22]. In short, purification was carried out through three chromatographic steps: an ion exchange on HiTrap Q FF at pH 9.5 with gradient elution, followed by two gel-filtration steps on Superdex 200 and Superdex 75 columns, on an AKTA Avant (GE Healthcare, Little Chalfont, UK) FPLC system. The purity of the proteins was checked by SDS-PAGE and was found to be at least 98%.

### 3.3. Wide-Line NMR

We have reviewed the varieties of radiofrequency excitations applied in wide-line NMR in two book chapters [23,24], and here we only address the simplest excitation protocol that uses a 90° (π/2) radiofrequency pulse. NMR measurements and data acquisition were performed with a Bruker AVANCE III NMR pulse spectrometer at a frequency of 82.4 MHz with a stability of better than $10^{-6}$. The π/2 pulse was 3–4 µs, and the dead time of the spectrometer was 6–8 µs. The inhomogeneity of the magnetic field was 2 ppm. The accumulated repeat number of the measurements was between 50 and 80.

The temperature was controlled by an open-cycle Janis cryostat with an uncertainty better than 0.5 K. The system was complemented by an adequate NMR head and by a closed sample holder.

## 4. Conclusions

By reinterpreting our previous results, we have determined the energy distribution of the potential barriers inhibiting the movement of water molecules bound to two protein molecules in aqueous solution. Based on our results, we could deduce quantitative conclusions about the ratios of the globular/ordered and more solvent exposed/disordered regions of the protein molecules and the extent of the latter, as well as the energy relations of the protein–water bonds. We suggest that short range forces (H-bonds) play a dominant role in the formation of the first hydrate shell.

The mapping of the water-binding characteristics of protein molecules is certainly not the only area of the application of wide-line NMR measurements and this novel interpretational procedure. The rapid, non-disruptive measurement and the data interpretation had already opened a novel avenue to study molecular interactions and to determine the moisture content of solid phase samples. In the outline of our previous work [9], we listed some additional possibilities. Three of these are also mentioned here: (i) the possibility to directly demonstrate the interaction between different molecules (e.g., protein and drug), (ii) the possibility of direct, non-destructive measurement of the different bonds between identical molecules, and (iii) the possibility to determine the effect of a standard (often NaCl containing) solvent on the structure and properties of protein molecules.

## Figures and Tables

**Table 1:** Characteristic thermal quantities for two sample proteins. *T*_fno_ end *T*_fne_ give the start and the end points of the plateau in *MD*s, respectively, as normalized fundamental temperature. *n*_ho_ and *n*_he_ values are given as the mobile hydration water fraction and as the number of mobile hydration water per protein molecule. *HeR*, *HeR*_n_, and *HeM* are dynamic parameters describing heterogeneity from various aspects (see text).

Protein	*T*_fno_	*T*_fne_	*HeR* (4) *	*n*_ho_	*n*_he_ **	*HeR*_n_ (6) *	*HeM*
UBQ	0.832 (4)	0.961 (5)	0.23 (2)	0.019 (1) 226 (3)	>0.009 (3) >102 (33)	0.3 (1)	241 (147)
ERD10	0.835 (3)	0.889(2)	0.73 (4)	0.0157 (4) 514 (13)	>0.098 (8) >3200 (275)	0.9 (1)	415 (60)

* The number in parentheses is the measurement error in the order of magnitude of the last number; the heterogeneity ratio is defined by the relation (4) or (6); ** Lower limit estimate due to the uncertainty of measured data is close to *T*_fn_ = 1; at *T*_fno_ value given in Table 1 (−43 °C), the excitation energy is 5.06 (4) kJ/mol for both proteins; at *T*_fne_ for ubiquitin, the excitation energy is 5.798 (2) kJ/mol at −9.9 °C; and for ERD10, it is 5.31 (3) kJ/mol at −36 °C.

## Abbreviations

FID	free induction decay
IDP	intrinsically disordered protein
MD	melting diagram
DMD	differential form of the melting diagram
DSC	differential scanning calorimetry
